# Le décollement épiphysaire fémoral supérieur post infectieux, à propos de deux cas

**DOI:** 10.11604/pamj.2014.18.319.2242

**Published:** 2014-08-21

**Authors:** Karima Atarraf, Mounir Arroud, Lamiae Chater, Moulay Abderrahmane Afifi

**Affiliations:** 1 Service d'Orthopédie Pédiatrique, Faculté de médecine et de pharmacie, Université Sidi Mohammed Ben Abdullah, CHU Hassan II, Fès, Maroc

**Keywords:** Décollement épiphysaire, fémur, post infectieux, epiphyseal detachment, femur, post infectious

## Abstract

Le décollement épiphysaire post infectieux est une pathologie très rare, jamais décrite dans la littérature. Nous rapportons deux cas colligés au service: le 1^er^ concerne un garçon âgé de 3 ans, admis pour impotence fonctionnelle du membre inferieur droit. La radiographie a objectivé un glissement de l’épiphyse fémorale supérieure par rapport à la métaphyse avec un épanchement échogène intra articulaire à l’échographie. Une artrotomie avec drainage ont été réalisé, puis le malade a été mis sous antibiothérapie et traction. L’évolution clinique a été bonne. Le 2^ème^ cas est celui d'un garçon âgé de 12 ans, admis pour prise en charge d'une boiterie gauche évoluant dans un contexte fébrile. La radiographie a objectivé un glissement de l’épiphyse fémorale supérieure par rapport à la métaphyse, l’échographie a montré un épanchement intra articulaire gauche. L’évolution a été marquée par la régression du syndrome infectieux après l'arthrotomie de la hanche et l'antibiothérapie.

## Introduction

Le décollement épiphysaire post infectieux et l'ostéomyélite du bassin sont deux entités pathologiques rares. Il complique le plus souvent une pathologie ostéo-articulaire non ou mal traitée. Nous rapportons deux cas d’épiphysiolyse fémorale supérieure septique dont l'une est survenue suite à une arthrite de la hanche, et l'autre secondaire à l'association d'une arthrite de la hanche à une ostéomyélite de l'os iliaque.

## Patients et observations

Nous avons réalisé la revue de deux dossiers de décollement épiphysaire post infectieux colligée au service d'orthopédie pédiatrique du CHU Hassan II, de Fès, sur une période de 04 ans avec un recul moyen de 02 ans.

### Observation N° 1

**Figure 1:**
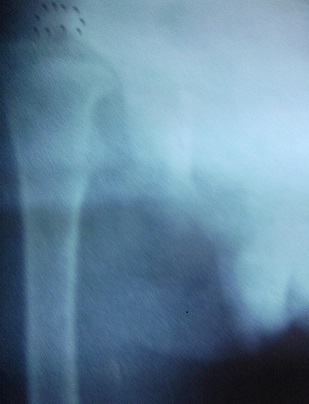
Décollement épiphysaire fémoral supérieur

Enfant de sexe masculin, âgé de 3 ans, adressé dans notre formation pour prise en charge d'une impotence fonctionnelle totale du membre inferieur droit évoluant depuis 03 semaines. L'examen clinique a trouvé un enfant léthargique, fébrile, déshydraté avec un flessum marqué de la hanche droite qui a été également tuméfiée en plus de l'impotence fonctionnelle totale. La radiographie du bassin a objectivé un glissement de l’épiphyse fémorale supérieure par rapport à la métaphyse (Figure 1) avec des images d'ostéolyse de l'aile iliaque. Un complément échographique de la hanche a montré un épanchement échogène intra articulaire avec infiltration des parties molles.

Le bilan inflammatoire a été très élevé avec une Vitesse de Sédimentation à 48mm la 1^ère^ heure et une hyperleucocytose à 33000 /mm à prédominance Polynucléaires neutrophiles. L'enfant a bénéficié d'une arthrotomie de la hanche qui a ramené un liquide purulent et a confirmé le décollement épiphysaire, avec un os iliaque friable de surface irrégulière, qui a été biopsié. La bi-antibiothérapie parentérale probabiliste à base de Céphalosporine de 3éme Génération a été démarrée puis rectifiée ultérieurement dés isolement d'un staphylocoque pathogène. L’étude histologique est revenue en faveur d'une ostéomyélite de l'aile iliaque. L'apyrexie a été obtenue dans les 48 heures, et la normalisation du bilan inflammatoire le 5^ème^ jour. L’évolution a été marquée par la destruction de la tête fémorale et par l'inégalité de longueur des membres inferieurs qui seront sujet d'une correction ultérieure.

### Observation n° 2

Enfant de sexe masculin, âgé de 12 ans, sans antécédents pathologiques particuliers. Qui a présenté depuis un mois une boiterie gauche évoluant dans un contexte fébrile, qui s'est compliquée par une impotence fonctionnelle totale du même membre. L'examen clinique a trouvé un enfant en mauvais état général, fébrile à 39° avec un teint grisâtre. L'examen locomoteur a trouvé un flessum de la hanche gauche avec limitation des mouvements de la hanche notamment l'abduction et la rotation interne. Le bilan biologique a montré une hyperleucocytose à 21000/mm^3^. Le bilan Inflammatoire était très élevé (CRP à 180ng/l et la VS à 80mm la 1^ère^ heure).

**Figure 2:**
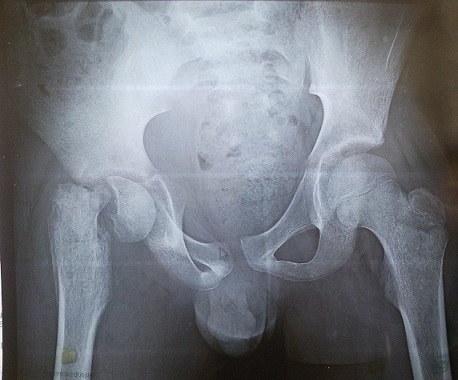
Décollement épiphyso-métaphysaire de l'extrémité supérieure du fémur

La radiographie du bassin de face a objectivé un glissement de l’épiphyse fémorale supérieure par rapport à la métaphyse (Figure 2). L’échographie articulaire a montré un épanchement intra articulaire de l'articulation coxo femorale gauche. Une arthrotomie de la hanche a été réalisée par voie de hueter modifiée qui a objectivé la présence de pus franc, avec un décollement métaphyso-épiphysaire pure de la tête fémorale. Le malade a bénéficié d'une traction collée avec prescription d'une antibiothérapie à base de pénicilline M et d'aminosides. L’étude cyto-bactériologique a objectivé un staphylocoque doré. L'apyrexie a été obtenue dans les 48 heures du post opératoire et l’évolution a été marquée par la raideur de la hanche. La chirurgie correctrice est prévue dès stérilisation des prélèvements bactériologiques.

## Discussion

Le décollement épiphysaire post infectieux est une pathologie très rare, jamais décrite dans la littérature. Pathologie souvent liée à la sous médicalisation et au sous diagnostic de certaines pathologies infectieuses. Survenant au niveau de l'extrémité supérieur du fémur, l’épiphysiolyse septique est le plus souvent secondaire à une ostéo- arthrite négligée de la hanche ou bien à une ostéomyélite entrainant une atteinte du cartilage métaphyso-épiphysaire. Son association à une ostéomyélite du bassin notamment de l'aile iliaque est peu décrite. D′un point de vue physiopathologique, pour Beaupré [1], il existe deux types d'ostéomyélite du bassin: Les ostéomyélites pré pubertaires qui surviennent entre 6 et 12 ans, étant le cas de l'un de nos malades et les ostéomyélites post pubères qui surviennent entre 12 et 18 ans.

Le tableau clinique du décollement post infectieux fait partie intégrante le plus souvent du tableau de la pathologie causale; qui le plus souvent pauvre; ce qui amène au retard diagnostic. Il s'agit d'une boiterie avec fièvre au long court. L'examen clinique et la biologie n'ont rien de particulier; ils rejoignent celui de la pathologie en cause. Le bilan radiologique peut objectiver le décollement épiphysaire pure métaphyso-épiphysaire, en plus des modifications de la structure osseuse et l′apparition d′une apposition périostée. L’échographie permet d'objectiver des collections abcédées ou bien un épanchement intra articulaire. L'IRM est d'un grand apport, C′est une exploration très sensible mais peu spécifique; elle apporte des renseignements anatomiques précis sur la topographie et l′étendue des lésions. Les séquences employées sont les séquences classiques d′examen ostéo-articulaire, elle n'a été réalisée chez aucun de nos malades par manque de moyens.

Le seul intérêt de la tomodensitométrie (TDM) réside à l′heure actuelle dans la recherche de séquestres osseux dans les formes chroniques d′ostéomyélite. La scintigraphie reste d'un intérêt majeur, Elle est peu irradiante, Sa sensibilité est grande, mais il existe des faux négatifs en cas de thrombose vasculaire étendue, ce qui reste une éventualité exceptionnelle. Par contre, Le foyer infectieux se traduisant par une hyperfixation ne présente aucun caractère de spécificité. Le drainage chirurgical pour évacuer d’éventuelles collections s'impose, ainsi qu'une arthrotomie pour un lavage de l'articulation. Le germe le plus souvent isolé est le staphylocoque doré dans plus de 90% des cas [1–4]. Un streptocoque peut être isolé, plus rarement un hemophilus influenzae qui est beaucoup plus fréquent dans les arthrites, Exceptionnellement, un bacille Gram négatif, une tuberculose [5], une infection mycosique. L'antibiothérapie visant le staphylocoque est débutée dans l'attente d'une identification du germe. Le pronostic de l’épiphysiolyse septique est sombre car pourvoyeuse d'ostéo- nécrose septique de la tête fémorale, ainsi que des troubles de croissance d'autant plus qu'ils surviennent sur un cartilage fertile.

## Conclusion

Le décollement épiphysaire post infectieux ou septique est une situation rare et grave, son pronostic reste incertain. Malgré le traitement, l’évolution se fait souvent vers des séquelles telles que la nécrose de la tête fémorale en plus des troubles de croissance, d'ou la nécessité de la prévention qui passe par le dépistage et le traitement des infections ostéo- articulaires. La palpation des repères osseux au niveau du bassin au cours de l'examen physique de tout enfant présentant une boiterie fébrile est capitale afin de détecter une éventuelle ostéomyélite pelvienne. L'IRM est d'un grand apport pour le diagnostic précoce de l'ostéomyélite; elle permet aussi de poser l'indication chirurgicale pour évacuer une éventuelle collection.
